# *Bdellovibrio bacteriovorus* HD100 guards against *Pseudomonas tolaasii* brown-blotch lesions on the surface of post-harvest *Agaricus bisporus* supermarket mushrooms

**DOI:** 10.1186/1471-2180-14-163

**Published:** 2014-06-20

**Authors:** Emma B Saxon, Robert W Jackson, Shobita Bhumbra, Tim Smith, R Elizabeth Sockett

**Affiliations:** 1 Genetics, School of Life Sciences, University of Nottingham, Medical School, Queen’s Medical Centre, Nottingham NG7 2UH, UK; 2 School of Biological Sciences, University of Reading, Whiteknights, Reading RG6 6AJ, UK

## Abstract

**Background:**

*Pseudomonas tolaasii* is a problematic pathogen of cultured mushrooms, forming dark brown ‘blotches’ on mushroom surfaces and causing spoilage during crop growth and post-harvest . Treating *P. tolaasii* infection is difficult, as other, commensal bacterial species such as *Pseudomonas putida* are necessary for mushroom growth, so treatments must be relatively specific.

**Results:**

We have found that *P. tolaasii* is susceptible to predation *in vitro* by the δ-proteobacterium *Bdellovibrio bacteriovorus.* This effect also occurred *in funga*, where *B. bacteriovorus* was administered to post-harvest mushroom caps before and after administration of the *P. tolaasii* pathogen. A significant, visible improvement in blotch appearance, after incubation, was observed on administration of *Bdellovibrio*. A significant reduction in viable *P. tolaasii* cell numbers, recovered from the mushroom tissue, was detected. This was accompanied by a more marked reduction in blotch severity on *Bdellovibrio* administration. We found that there was in some cases an accompanying overgrowth of presumed-commensal, non-*Pseudomonas* bacteria on post-harvest mushroom caps after *Bdellovibrio-*treatment. These bacteria were identified (by 16SrRNA gene sequencing) as *Enterobacter* species, which were seemingly resistant to predation. We visualised predatory interactions occuring between *B. bacteriovorus* and *P. tolaasii* on the post-harvest mushroom cap surface by Scanning Electron Microscopy, seeing predatory invasion of *P. tolaasii* by *B. bacteriovorus in funga.* This anti-*P. tolaasii* effect worked well in post-harvest supermarket mushrooms, thus *Bdellovibrio* was not affected by any pre-treatment of mushrooms for commercial/consumer purposes.

**Conclusions:**

The soil-dwelling *B. bacteriovorus* HD100 preys upon and kills *P. tolaasii*, on mushroom surfaces, and could therefore be applied to prevent spoilage in post-harvest situations where mushrooms are stored and packaged for sale.

## Background

*Pseudomonas tolaasii* is a Gram-negative, naturally soil-dwelling bacterial pathogen that causes brown blotch disease in several varieties of cultivated mushrooms [1-3]. The disease is characterised by brown lesions on the outer layers (2–3 mm depth) of the mushroom pileus and stipe, which range from small, light brown spots to larger, dark, sunken and wet lesions, depending on disease severity. This brown discolouration results from mushroom production of melanin, which is a defence response induced in this case by *P. tolaasii* producing the toxin tolaasin. Tolaasin is an 18-amino acid lipodepsipeptidide that forms ion channels and also acts as a biosurfactant to disrupt the plasma membrane of mushroom cells, allowing *P. tolaasii* access to cell-nutrients [4-7]. Infection is also reported to result in slower development of the mushroom crop with a lower yield [8]. The economic impact of the disease is significant, resulting in loss of visual appeal to consumers and regular crop reductions of 5–10% in the UK [9]. The disease is found worldwide: *P. tolaasii* mushroom infection has been documented in several countries, including the USA, Spain, Serbia, the Netherlands, Japan and Korea [1,2,10-13].

A major obstacle in the control of *P. tolaasii* infection that contributes to its broad prevalence is that some of the bacterial species present in the casing soil around mushrooms, such as *Pseudomonas putida*, are necessary for promoting the initial stages of mushroom growth [14,15]. This means that the casing soil cannot be sterile, and broad range antibiotic and antiseptic treatments cannot be used in the mushroom-growing process; consequently, *P. tolaasii* may become endemic in the casing soil and compost used in mushroom cultivation [16].

*P. tolaasii* survives well in nutrient-poor environments, such as the casing soil prior to mushroom growth, by altering the production of various enzymes, thus switching between pathogenic non-fluorescent (Smooth colony morphology on King’s Medium B agar, S-type) and non-pathogenic fluorescent (Rough colony morphology, R-type) forms [17,18]. *P. tolaasii* also uses flagellar-mediated chemotaxis in the wet casing soil to move towards nutrient ‘signals’ produced by the mushroom; once on the pileus surface, they attach and initiate disease rapidly [5,19]. Symptoms can appear on mushrooms at all stages of development; some apparently unaffected mushrooms also develop symptoms after harvesting, making it difficult to immediately identify and target *P. tolaasii* infections [20]. Furthermore, the pathogen is spread easily on the hands of mushroom pickers, and epidemics can occur between multiple mushroom houses [8].

Due to the adaptability and persistence of *P. tolaasii*, and the limitations on treatment options, there are very few effective methods for controlling *P. tolaasii* infection that are also safe to use on crops intended for human consumption. The current best methods of disease prevention are addition of chlorinated compounds such as calcium hypochlorite to irrigation water, and careful control of growth conditions; for example, the surface moisture of mushrooms and water level in the casing soil to minimize *P. tolaasii* chemotaxis and motility; however, the success of disease prevention is highly variable, and not guaranteed [8,13,21].Other disinfectants and antibiotic compounds such as chloramine T and bronopol have been suggested as potential treatments [13,22], as well as natural plant extracts from *Salvia miltiorrhiza*[23], and the White Line Inducing Principle (WLIP) produced by *Pseudomonas reactans*, which reacts with tolaasin produced by *P. tolaasii*[24]. Other Pseudomonads that are antagonistic to *P. tolaasii*, such as *Pseudomonas flourescens*, have also been investigated as biocontrol strains [25]. Most recently, the application of a *P. tolaasii*-specific bacteriophage has been proposed as a novel method of controlling *P. tolaasii* infection [26], but to our knowledge none of these alternative disease prevention methods have been tested or used commercially.

The Gram-negative predatory bacterium *Bdellovibrio bacteriovorus* has been discussed as a potential ‘living antibiotic’ for bacterial pathogens of humans and animals. *Bdellovibrio* attach to, invade and replicate inside diverse Gram-negative bacterial prey, killing the prey cell in the process (For more detail, see [27,28]). *Bdellovibrio* was isolated from soil, and thus evolved to kill bacterial prey in soil and water [29]. It is highly motile in liquid, using flagellar swimming [30], and it also ‘glides’ slowly on solid surfaces [31], and uses chemotaxis to locate regions rich in prey [32]. Despite thus being an ideal candidate for the treatment of crop pathogens, the influence of *Bdellovibrio* predation on Gram-negative disease outbreaks in the soil environment remains largely unknown. The effect of *Bdellovibrio* on Gram-negative bacterial pathogen populations has previously been studied in live chickens and on soybean plant leaves rubbed into scratches made artificially on leaf tissue [33,34]. The supply of *Bdellovibrio bacteriovorus* HD100 orally to live chickens showed that, while they did reduce pathogen numbers and alter the gut microbiota, there were not any harmful effects of ingestion of *Bdellovibrio*, which is important in a food-related setting [33].

In this current study, we investigated whether *Bdellovibrio* can be used to control the soil-borne mushroom pathogen *P. tolaasii* in the natural environment of the surface of the cultivated button mushroom *Agaricus bisporus* post-harvest. We measured the effect of *Bdellovibrio bacteriovorus* HD100 application on the extent of brown blotch lesion symptoms resulting from *Pseudomonas tolaasii* 2192^T^ inoculation onto mushroom pilei, and compared these with *P. tolaasii* cell counts recovered from inoculated mushrooms. We also monitored the interaction between *B. bacteriovorus* HD100 and *P. tolaasii* 2192^T^ on the mushroom pileus surface to confirm *Bdellovibrio* predation of the pathogen *in funga*. Bacterial-fungal interactions have been the subject of recent reviews [35] as they involve interesting cross kingdom biology, but also affect crop productivity and thus global food security. In this study, a bacterial-bacterial interaction on a fungal surface prevents a pathogenic bacterial-mushroom interaction through an active, predatory process, rather than displacement by competition, which is the first time this has been documented.

## Results

### *Bdellovibrio* inhibits *P. tolaasii* population growth *in vitro*

**Figure 1:**
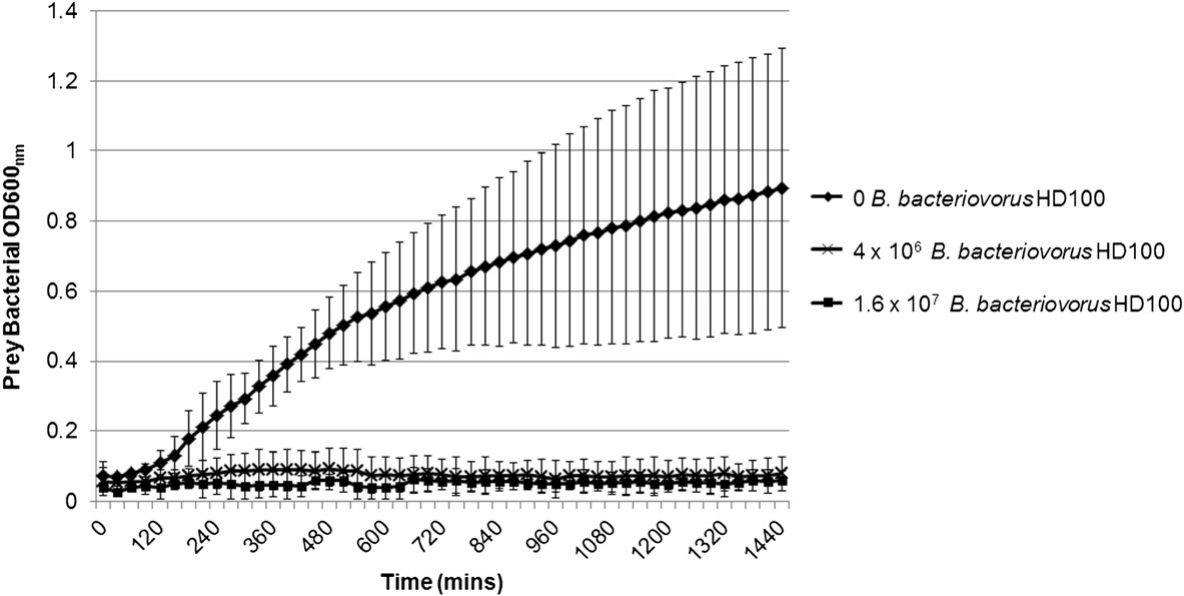
**Reduction in *P. tolaasii *OD600**_**nm **_**over 24 hours, *in vitro*, in the presence of *Bdellovibrio bacteriovorus*.** Mean OD600_nm_ of *P. tolaasii* 2192^T^ samples in the absence or presence of live *B bacteriovorus* HD100 added at 4 × 10^6^ or 1.6 × 10^7^ Plaque Forming Units (PFU) (n = 4). The increase in OD600_nm_ in the absence of *Bdellovibrio* indicates *P. tolaasii* 2192^T^ growth, while no increase in the presence of 4 × 10^6^ or 1.6 × 10^7^*B. bacteriovorus* HD100 indicates inhibition of *P. tolaasii* 2192^T^ growth. Error bars indicate 95% Confidence Intervals for each OD600_nm_ value.

To begin to test *Bdellovibrio* as a possible biocontrol agent against *P. tolaasii*, we first aimed to assess the impact of their co-incubation on *P. tolaasii* survival *in vitro*. As Figure 1 shows, The Optical Density (OD600_nm_) of *P. tolaasii* 2192^T^ samples in the presence of live *B. bacteriovorus* HD100 did not increase compared to a heat-killed *B. bacteriovorus* HD100 control, measured over 24 hours in the BMG plate-reader. (*Bdellovibrio* cells alone are too small to produce an OD600_nm_ reading). In the presence of *B. bacteriovorus* HD100 at both 4 × 10^6^ cells/well and 1.6 × 10^7^ cells/well, the OD600_nm_ of *P. tolaasii* 2192^T^ did not increase from the starting value (OD600nm = 0.05, 9.7 × 10^6^ CFU/well) over 24 hours. However, when live *B. bacteriovorus* HD100 were substituted with heat-killed *B. bacteriovorus* HD100, the OD600_nm_ value increased from 0.08 to a final value of 0.89, corresponding to an increase from 1.6 × 10^7^ to 1.7 × 10^8^ CFU over 24 hours, (n = 3, Figure 1). This indicates that *Bdellovibrio* effectively suppressed the population growth of *P. tolaasii*, most likely due to killing by predation.

### Brown blotch lesion intensity was reduced by *Bdellovibrio* application onto mushrooms

**Figure 2:**
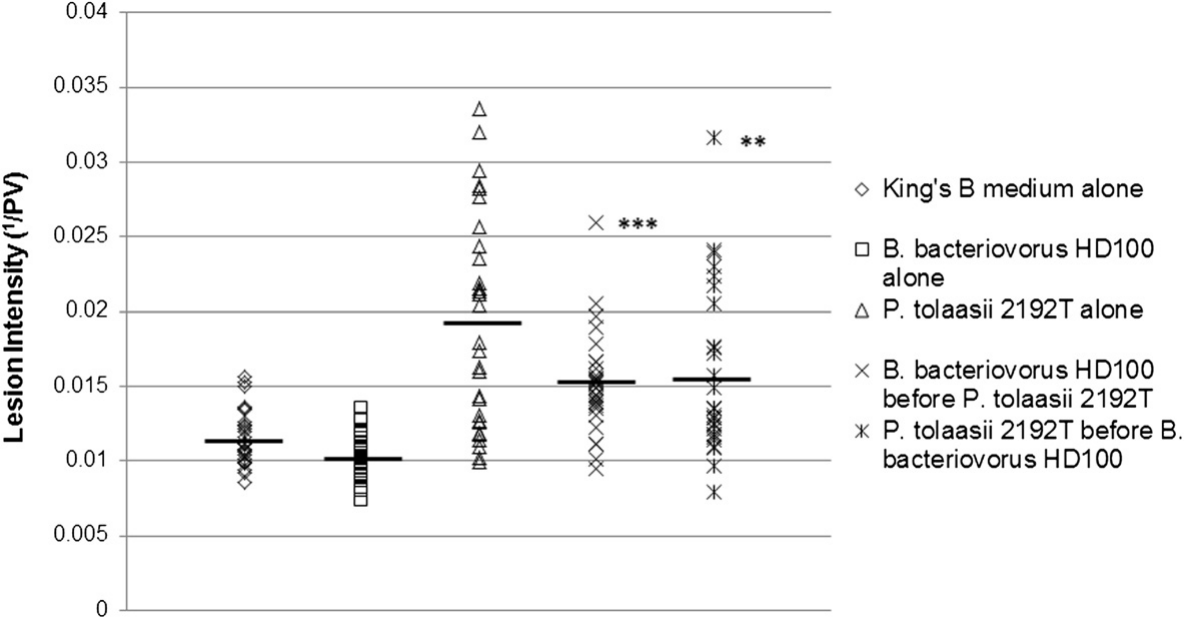
**Lesion intensity on *P. tolaasii*-inoculated mushrooms in the presence and absence of *Bdellovibrio*.** Lesion intensities on mushroom pilei under 5 different treatment conditions, detailed to the right of the graph. Each *P tolaasii* 2192^T^ inoculation contained 1.7 × 10^6^ CFU, and each *B. bacteriovorus* HD100 inoculation contained 2.9 × 10^6^ PFU*.* Higher lesion intensity indicates a greater level of brown blotch disease symptoms and therefore a higher level of *P. tolaasii* infection. Horizontal black bars indicate the mean lesion intensity value for each treatment group. Student’s t-test of significance between *B. bacteriovorus* HD100 treated and non-treated mushrooms inoculated with *P. tolaasii* 2192^T^: **p < 0.01, ***p <0.001.

Given *B. bacteriovorus* HD100 was observed to suppress *P. tolaasii* 2192^T^ growth in vitro, we reasoned that this effect might be replicated in a more natural environment. We first aimed to determine whether symptoms of *P. tolaasii* infection, a function of bacterial metabolism and growth, were reduced with *Bdellovibrio* treatment in a natural context*.* The intensity of lesions formed by *P. tolaasii* 2192^T^ on the post-harvest pileus surface of the cultivated button mushroom *Agaricus bisporus* was measured in the presence and absence of *B. bacteriovorus* HD100*,* as shown in Figure 2*.* Mushroom pilei inoculated with *P. tolaasii* 2192^T^ alone, in the absence of any treatment with *B. bacteriovorus* HD100, formed dark, wet surface lesions, the primary symptom of brown blotch disease, after 48 hours at 29°C (mean intensity = 0.019 ^1^/PV ± 0.0005, n = 30). In contrast, pilei treated with a King’s Medium B control (the preferred growth medium of *P. tolaasii*) did not form these dark lesions (mean intensity = 0.012 ^1^/PV ± 0.0005, n = 30); similarly, those treated with *B. bacteriovorus* HD100 alone, and not inoculated with *P. tolaasii* 2192^T^, also did not form dark lesions (mean intensity = 0.010 ^1^/PV ± 0.0005, n = 30), so *Bdellovibrio* application itself did not have a significant adverse effect on the appearance of mushroom pilei.

Post-harvest mushrooms treated with *B. bacteriovorus* HD100 either 30 minutes before or 30 minutes after *P. tolaasii* 2192^T^ inoculation developed significantly lighter lesions than those inoculated with *P. tolaasii* 2192^T^ alone (average intensity = 0.015 and 0.016 1/PV ± 0.0005 respectively, n = 30 in both cases, vs. 0.019 1/PV ± 0.0005 for mushrooms inoculated with *P. tolaasii* 2192^T^ alone). This demonstrates that *Bdellovibrio* effectively reduces the dark lesions of brown blotch disease caused by *P. tolaasii*, and that this reduction is slightly greater where *Bdellovibrio* is added before *P. tolaasii*. The significance of the difference in lesion intensities between *B. bacteriovorus* HD100 treated and untreated, *P. tolaasii* 2192^T^ inoculated mushrooms was greater when *Bdellovibrio* was added before *P. tolaasii* 2192^T^ than when added after (Student’s t-test p < 0.001 for *B. bacteriovorus* HD100 added before *P. tolaasii* 2192^T^ vs. *P. tolaasii* 2192^T^ alone, p < 0.01 for *B. bacteriovorus* HD100 added after *P. tolaasii* 2192^T^ vs. *P. tolaasii* 2192^T^ alone). *Bdellovibrio* application may therefore be more effective as a preventative measure to protect mushrooms against brown blotch disease, rather than a treatment for an already infected mushroom crop, and could be explored as a background addition to mushroom compost or casing layers to maintain “health”.

### Scanning Electron Microscope images show *B. bacteriovorus* attachment and bdelloplast formation in *P. tolaasii* cells

**Figure 3:**
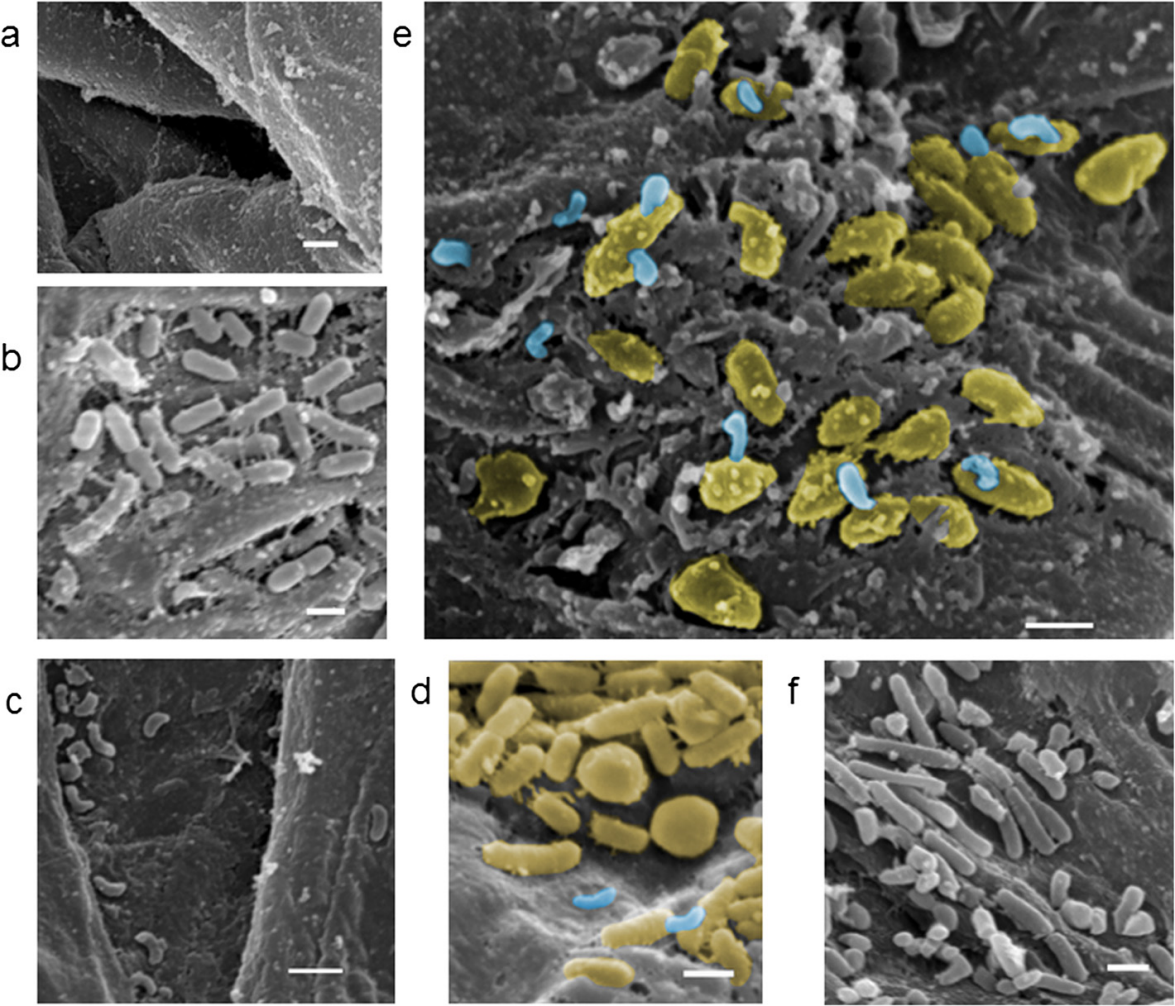
**Predatory interactions between *Bdellovibrio *and *P. tolaasii *“*in funga” *on the mushroom pileus surface.** Scanning Electron Microscope images showing the mushroom pileus surface 48 hours after the following treatments: **a**. untreated mushroom pileus surface **b**. inoculation of *P. tolaasii* 2192^T^ alone **c**. Inoculation of *B. bacteriovorus* HD100 alone **d**. and **e**. Co-inoculation of *P. tolaasii* 2192^T^ and *B. bacteriovorus* HD100 and **f**. Application of King’s medium B alone. In image **e**, *B. bacteriovorus* HD100 (blue) are shown attached at one pole to *P. tolaasii* 2192^T^ (yellow), a crucial first step in the predatory process. Images **d** and **e** both show rounded *P. tolaasii* 2192^T^ cells, characteristic of the bdelloplast structures formed after *Bdellovibrio* invades the host cell and begins replication. 1 μm scale bar shown.

To confirm whether the reduction in *P. tolaasii* 2192^T^ numbers and brown blotch lesion intensity was due to *B. bacteriovorus* HD100 predation *in funga* or another competition for resources, the interaction between *P. tolaasii* and *Bdellovibrio* was monitored in samples from the surface of the post-harvest *A. bisporus* (shown untreated in Figure 3a), 48 hours after mushroom treatments, using Scanning Electron Microscopy (SEM). *P. tolaasii* 2192^T^ added alone to the mushroom pileus accumulated together, in an arrangement parallel to the pileus surface, in the pits present between chitin fibres (Figure 3b). Fibrillar structures attached to the *P. tolaasii* 2192^T^ cells were frequently observed, which have also been documented in previous microscopic studies [36]. These resemble pili, with extracellular polymeric substances laid down on them, and may allow *P. tolaasii* to adhere tightly to the mushroom surface and to each other in a biofilm, to rapidly initiate disease (Figure 3b [37]). *B. bacteriovorus* HD100 added alone to the mushroom surface survived after 48 hours and also accumulated in the small pits between chitin fibres (Figure 3c).

Where *B. bacteriovorus* HD100 was added to the mushroom surface both before (Figure 3e) and after *P. tolaasii* 2192^T^ (Figure 3d), *B. bacteriovorus* HD100 attachment to *P. tolaasii* 2192^T^ cells was observed: a crucial first step in the predatory process. In addition, bdelloplasts, the rounded, dead *P. tolaasii* structures in which *Bdellovibrio* establish, grow and replicate after attachment and invasion, were also observed where *B. bacteriovorus* HD100 was added before or after *P. tolaasii* 2192^T^. Although a valid statistical survey is not possible in these SEM samples, bdelloplasts were most clearly visible on the mushroom surface where *B. bacteriovorus* HD100 was added before *P. tolaasii* 2192^T^ (Figure 3d). This correlates with the greater reduction in lesion intensity measurements on mushrooms where *B. bacteriovorus* HD100 was added before *P. tolaasii* 2192^T^ (Figure 2): *Bdellovibrio* attachment to prey and subsequent bdelloplast formation may be easier, and occur more rapidly, where *P. tolaasii* cells have not had time to accumulate, adapt and adhere to the mushroom surface, preventing *P. tolaasii* from producing as much tolaasin, and thus reducing the extent of the characteristic brown blotch symptoms.

A King’s Medium B control addition to the pileus resulted in the growth of different types of bacterial cells, with different morphologies that were distinct from that of *P. tolaasii* 2192^T^ & *B. bacteriovorus* HD100 (Figure 3f); however, typically, no bacterial cells were observed on untreated mushroom tissue (Figure 3a). This indicates that the supermarket mushrooms carry a small, indigenous bacterial microflora that replicates readily in added growth medium, which may impact upon *P. tolaasii* CFU numbers recovered from experimentally inoculated tissue, as described below.

### Application of *Bdellovibrio* before inoculation with *P. tolaasii* reduced the number of *P. tolaasii* in infected mushroom tissue

**Figure 4:**
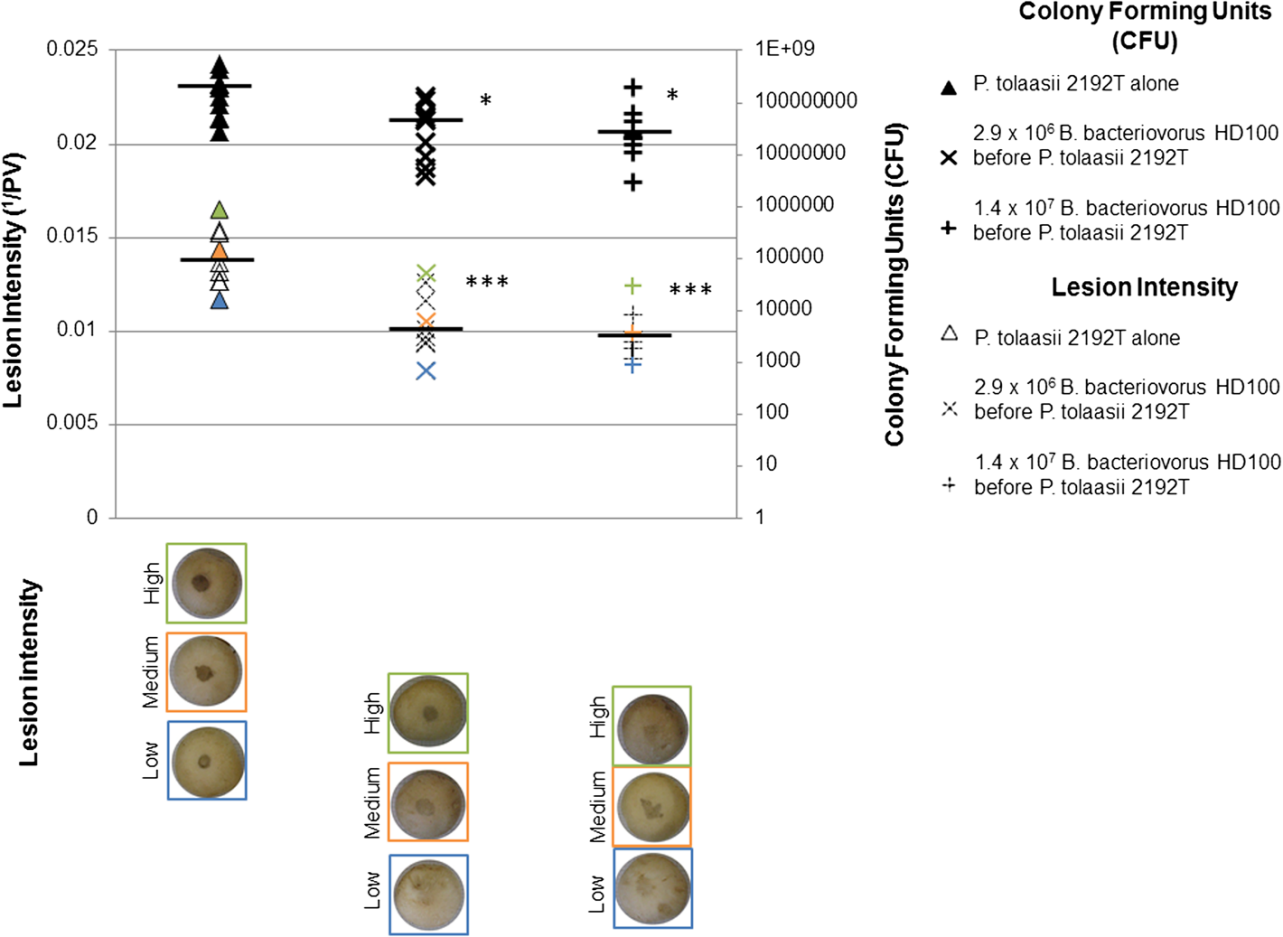
**Bacterial CFU numbers recovered from *P. tolaasii*-inoculated mushrooms in the presence and absence of *Bdellovibrio*.** Lesion intensities and number of bacterial colony forming units (CFU) recovered from mushroom pilei subject to three different treatments detailed to the right. Each *P. tolaasii* 2192^T^ inoculation contained 1.7 × 10^6^ CFU. Images of mushrooms with typical: high, mean, and low intensity lesions in each group are shown below the graph. Horizontal black bars indicate the mean values for lesion intensity/CFU count in each treatment group. Student’s t-test of significance between *B .bacteriovorus*-treated and non-treated mushrooms inoculated with *P. tolaasii* 2192^T^: *p <0.05, ***p <0.001.

To determine whether the reduction in lesion intensity after treatment with *B. bacteriovorus* HD100 correlated with a reduction in *P. tolaasii* 2192^T^ cell numbers, CFU were recovered and enumerated from mushroom tissue that had been inoculated with *P. tolaasii* 2192^T^ and pre-treated with *B. bacteriovorus* HD100*,* compared with a *P. tolaasii* 2192^T^ inoculated, non-*B. bacteriovorus* HD100 treated control (Figure 4). A mean number of 4.5 × 10^7^ and 3.9 × 10^7^ CFU were recovered from mushrooms pre-treated with 2.9 × 10^6^ or 1.4 × 10^7^ PFU live *B. bacteriovorus* HD100 respectively, which were both significantly lower than the mean 1.9 × 10^8^ CFU recovered from mushrooms inoculated with *P. tolaasii* 2192^T^ alone (Student’s t-test of difference p < 0.05); these observations correlated with a significant reduction in lesion intensity (p < 0.001) on mushrooms treated with 2.9 × 10^6^ and 1.4 × 10^7^ PFU *B. bacteriovorus* HD100 (mean = 0.010 ^1^/PV in both cases) compared with mushrooms inoculated with *P. tolaasii* 2192^T^ alone (mean = 0.014 ^1^/PV). Despite this significant reduction in lesion intensity, the total number of CFU recovered from *B. bacteriovorus* HD100 treated mushrooms onto King’s Medium B was high, suggesting that the bacteria recovered from seemingly similar, beige-coloured colonies on the King’s Medium B plates were not solely pathogenic *P. tolaasii* 2192^T^, but might include other species indigenous to the mushroom pileus surface that are not well preyed upon by *B. bacteriovorus* HD100, as observed in SEM images of mushroom tissue to which King’s medium B broth was added alone.

### *Enterobacter* species are present on the surface of some commercially produced supermarket mushrooms

The number of CFU recovered from the mushrooms that were treated with *B. bacteriovorus* HD100 after inoculation with *P. tolaasii* was relatively high compared to mushrooms inoculated with *P. tolaasii* alone. To confirm the identity of the bacteria seen in Figures 3d and e and recovered from supermarket mushroom tissue pre-treated with *B. bacteriovorus* HD100 before *P. tolaasii* 2192^T^ at both 2.9 × 10^6^ and 1.4 × 10^7^ PFU ml^−1^, 20 colonies taken from the King’s medium B agar plates used to enumerate bacterial CFU, recovered from the treated mushroom tissue of two mushrooms in each group, were grown on Coliform Chromogenic agar (oxoid). This agar contains two chromogenic substrates that turn purple when cleaved by the enzymes glucorinidase and galactosidase, which are both present in coliforms such as *E. coli*, and absent from Pseudomonads (including *P. tolaasii*); all 20 colonies were pigmented purple indicating them as coliform, closely related to *E. coli*, and therefore as indigenous species to the mushroom pileus, and distinctly different to *P. tolaasii* 2192^T^*,* which produced straw coloured colonies on the agar. Three of these coliform isolates were identified by 16 s rDNA sequencing as members of the *Enterobacter* genus using the BLAST online tool (http://blast.ncbi.nlm.nih.gov/Blast.cgi), which showed that the isolates were most closely related to *Enterobacter* sp. LB9 (GenBank: JQ864377.1) matching 99% identity. This explains the relatively high number of total bacterial colonies recovered from mushroom tissue treated with *Bdellovibrio*, despite the reduction in the dark lesions characteristic of *P. tolaasii* infection: *Bdellovibrio* predation rapidly reduces *P. tolaasii* population numbers on the mushroom surface, but does not necessarily reduce those of other non-disease causing, likely mushroom-indigenous species, such as the *Enterobacter* isolated in this study*.* The King’s Medium B in which *P. tolaasii* 2192^T^ and *B. bacteriovorus* HD100 were added to the surface of the mushroom during test inoculations, and the cell-lysate debris left behind after *P. tolaasii* death due to predation, may then allow these indigenous *Enterobacter* to occupy the niche caused by *Bdellovibrio* predation of *P. tolaasii*.

## Discussion

We showed that *B. bacteriovorus* HD100 is a predator of *P. tolaasii* 2192^T^*in vitro* and *in vivo* (*in funga*), suppressing population growth of the strain over a 24-hour period where 4 × 10^6^ or 1.6 × 10^7^ PFU *B. bacteriovorus* HD100 were added to pathogen on post-harvest mushrooms (Figures 1 and 4). *P. tolaasii* is a difficult pathogen to control in mushroom grow-houses due to its ability to persist in nutrient-poor soils and the ease with which it spreads through mushroom compost, through flagellar swimming, and via the hands of pickers during the manual harvesting process [8]. Furthermore, commensal bacterial species in the mushroom casing soil play a key role in mushroom growth initiation, and therefore any treatment to prevent or treat *P. tolaasii* infection must not result in a completely sterile growth environment, which may result from broad antibiotic or antiseptic treatment. Thus it is beneficial to explore post-harvest anti- *P. tolaasii* treatments, such as this study with *B. bacteriovorus.*

Our SEM images confirmed that *B. bacteriovorus* HD100 survived on the post-harvest supermarket mushroom surfaces after 48 hours, and was therefore unaffected by any pre-treatment of those mushrooms for commercial purposes to promote growth and extend shelf-life in the film-covered plastic trays they were sold in (Figure 3c). *B. bacteriovorus* is therefore a viable treatment for bacterial diseases of mushrooms, such as brown blotch disease. Previous studies of mushroom infections have found that a ‘threshold’ number of *P. tolaasii* cells are required for the initiation of infection, which includes production of tolaasin, the chemical mediator of the brown blotch symptom development [8]. We found that when *B. bacteriovorus* HD100 was applied to the surface of post-harvest, commercially grown mushrooms before or after inoculation with *P. tolaasii*, both the intensity of the brown blotch symptoms of disease and the number of *P. tolaasii* 2192^T^ present the mushroom surface were significantly reduced (Figures 2 and 4), supporting the threshold hypothesis. Our SEM images also showed that *B. bacteriovorus* HD100 attached to, invaded and killed *P. tolaasii* 2192^T^ cells by forming bdelloplasts on the pileus surface, when added both before or after *P. tolaasii* 2192^T^ inoculation (Figure 3d and e); thus, reduction in *P. tolaasii* 2192^T^ numbers and disease symptoms was due to predatory activity by *B. bacteriovorus* HD100. As the consumer preference is for white, clean-looking mushrooms with minimal surface damage, the reduction in brown blotch tissue damage by *B. bacteriovorus* application could increase the yield and possibly the shelf life of high-quality, marketable mushrooms. This study investigated the survival of *B. bacteriovorus* HD100 and its predatory activity against *P. tolaasii* on the surface of post-harvest mushrooms up to 48 hours, sufficient time for brown blotch disease to develop on untreated mushrooms. Thus studies over longer time points, covering time from transportation to the sell-by date, would need to be investigated, in future work, if *Bdellovibrio* was to be applied as a treatment to extend shelf-life.

In addition to reducing the population of *P. tolaasii* on the mushroom surface, *Bdellovibrio* are natural soil dwellers and so their application to casing soil could also prevent spread of brown blotch between mushrooms in the growth environment and between grow houses. In this way, the fast swimming motility of *Bdellovibrio*[38] would allow efficient location of *P. tolaasii* prey, using chemotaxis, in the wet casing soil prior to mushroom growth initiation, and translocation by gliding along the mushroom pileus surface after mushroom fruiting bodies have formed, preventing *P. tolaasii* infection establishment at multiple stages of mushroom growth; previously, the possibility of infection throughout the mushroom growth period has been an obstacle in brown blotch disease control. Further pre-harvest studies could investigate the longevity and protective effect of *Bdellovibrio* inoculated into the casing soil around mushroom mycelium, before and after fruiting body initiation, on growing *A. bisporus*. As *Bdellovibrio* preys efficiently upon some, but not all, species of *Pseudomonas* (unpublished observations), and some Pseudomonads in the casing soil such as *P. putida* are important in fruiting body initiation; further studies would additionally investigate the predatory activity of *B. bacteriovorus* HD100 against such commensal strains *in vitro* and in the casing soil to ensure that there are no effects that would have an adverse impact on mushroom fruiting body production.

As host-dependent *Bdellovibrio* require prey cells to survive, the post-harvest treatment could also be self-limiting, as *Bdellovibrio* would die once *P. tolaasii* prey had been eradicated; further studies could quantify this. Furthermore, these *in vitro* and *in vivo* predation studies suggest that *B. bacteriovorus* may be able to survive the action of the toxins produced by *P. tolaasii* and other members of the *Pseudomonas* genus, including tolaasin and other lipases and peptidases, which cause the damage to the mushroom pileus [39]. This suggests that *Bdellovibrio* species may be effective against other crop pathogenic bacterial species, even if they produce biologically active secreted compounds. This could be followed up with studies of the pure compounds themselves versus *B. bacteriovorus.*

We infrequently isolated *Enterobacter* species in our experiments from supermarket mushrooms, likely being commensals growing in number after pre-treatment with *B. bacteriovorus* HD100, suggesting that these *Enterobacter* isolates are not susceptible to *Bdellovibrio* predation. A Plant Growth Promoting (PGP) *Enterobacter* species, *Enterobacter cloacae,* has been described previously, which colonises rice root surfaces and competes with other species in the soil microbiota for nutrients [40]. Enterobacter species have also previously been isolated from spent mushroom compost [41], where they might associate with the mushroom surface in a similar way, competing with other mushroom-indigenous bacteria as commensal species. As *Bdellovibrio* has previously been shown to prey upon diverse *Enterobacter* species [42], it was unexpected that numbers seemed unaffected by *Bdellovibrio* predation; inhibition of predation in this case may be due to a factor such as the presence of a protective S-layer, which may prevent *Bdellovibrio* from attaching to and invading *Enterobacter* prey cells [43], but confirming S-layer presence was beyond the scope of this study. The *Enterobacter* species in this study were isolated from *Bdellovibrio*-treated mushroom tissue, unaffected by any brown blotch disease symptoms; and so the species are unlikely to be pathogenic, and may be commensals. It could therefore be beneficial that *Bdellovibrio* are unable to prey upon the *Enterobacter* species isolated in this study, preserving any beneficial commensal effect they might have, while still protecting against *P. tolaasii* infection.

## Conclusions

*Bdellovibrio bacteriovorus* HD100 are terrestrial bacteria which show natural control of *Pseudomonas tolaasii,* a spoilage pathogen of mushroom crops, on the non-sterile, biotic surface of the mushroom pileus. These terrestrial bacteria therefore have a natural ability to act as “food security guards” against Gram-negative crop pathogens.

## Methods

The bacterial strains and primers used in this study are listed in Tables 1 and 2, respectively.

**Table 1:** Bacterial strains used in this study

**Strain**	**Description**	**Reference**
*Escherichia coli* S17-1 (used as prey to initially culture *Bdellovibrio*)	*thi, pro, hsdR*^*−*^*, hsdM*^*+*^*, recA,* integrated plasmid RP4-Tc::Mu-Kn::Tn7	[44]
*Bdellovibrio bacteriovorus* HD100	Type strain, genome sequenced	[29,45]
*Pseudomonas tolaasii* 2192^T^	Type strain, NCPPB No. 2192^T^, brown blotch pathogen of mushrooms, acquired from RW Jackson (University of Reading)	[46]

**Table 2:** Primers used in this study

**Name**	**Sequence**	**Description**
16 s_8F	AGAGTTTGATCMTGGC	‘Universal’ forward primer designed to amplify/sequence diverse bacterial 16 s rDNA sequences [47]
16 s_1492rev	TACCTTGTTAYGACTT	‘Universal’ reverse primer designed to amplify/sequence diverse bacterial 16 s rDNA sequences [47]

### Bacterial culturing procedures

*E. coli* S17-1 was grown in YT medium (5 g/L Sodium Chloride, 5 g/L Peptone, 8 g/L Tryptone, pH 7.5) shaken at 200 rpm at 37°C for 16 hours. The predatory, host-dependent *B. bacteriovorus* HD100 was cultured at 29°C on *E. coli* S17-1 prey cells on YPSC medium agar (0.125 g/L Magnesium Sulphate, 0.25 g/L Sodium Acetate, 0.5 g/L Bacto Peptone, 0.5 g/L Yeast Extract, 0.25 g/L Calcium Chloride Dihydrate, pH 7.6) using an overlay plate technique. Liquid predatory cultures of *B. bacteriovorus* HD100 for predation tests were produced by 16 hour incubation at 29°C in 2 mM CaCl_2_ 25 mM HEPES pH 7.6 buffer, containing *E. coli* S17-1 prey, both methods described in detail elsewhere [30]. Following growth the *B. bacteriovorus* HD100 were filtered by passage twice through Millipore 0.45 μm syringe filters to remove any remaining prey. *P. tolaasii* 2192^T^ was grown in King’s Medium B (Prepared using Scientific Laboratory Supplies Bacto™ Proteose Peptone No. 3, product code 221693, according to the UNE-EN 12780 standard protocol, Cat. No. 1154) at 29°C for 16 hours. When isolating indigenous bacteria from mushrooms Coliform chromogenic agar (Oxoid, product code CM0956) was used, again with incubation at 29°C.

### *B. bacteriovorus* predation of *P. tolaasii* populations grown *in vitro*

*B. bacteriovorus* predation of *P. tolaasii* was firstly tested in a buffer-*Pseudomonas* King’s medium B suspension in a plate reader. 180 μl/well of a 50% v/v King’s Medium B, 50% v/v 2 mM CaCl_2_ 25 mM HEPES pH 7.6 buffer mixture was added to the wells of a clear-bottomed, 96-well Krystal microplate (Porvair Sciences Ltd, Product No. 215006). 1.5 ml aliquots of predatory cultures of *B. bacteriovorus* HD100, containing 2.5 × 10^8^ PFU ml^−1^, were prepared and heat killed at 105°C for 5 minutes and allowed to cool to ambient temperature (21°C). This heat-killed, cooled culture was then added, in a 3:1 ratio, to a live liquid culture of *B. bacteriovorus* HD100 to give 6.3 × 10^7^ PFU ml^−1^ of live *B. bacteriovorus* HD100. This was used as a diluted application of *Bdellovibrio* to achieve a lowered concentration of predator in our experiments. Microplate wells were then set up using either 64 μl of the heat-killed culture alone as a negative control; 64 μl of the heat-killed/live mixture described above; or 64 μl of the original live culture of *Bdellovibrio*. These preparations gave final live *B. bacteriovorus* HD100 cell numbers of 0, 4 × 10^6^ or 1.6 × 10^7^ PFU, respectively. For test prey cells, a liquid culture of *P. tolaasii* 2192^T^, containing 7.4 × 10^8^ CFU/ml^−1^, was diluted 2 in 5 to give 3.0 × 10^8^ CFU/ml^−1^ in 50% v/v King’s Medium B, 50% v/v 2 mM CaCl_2_ 25 mM HEPES pH 7.6 buffer mixture. 20 μl of this diluted *P. tolaasii* 2192^T^ containing 5.9 × 10^6^ CFU was transferred to the microplates containing the predator mixtures. The plates were then sealed with a Breatheasy® seal (Diversified Biotech Cat. No. BEM-1) and transferred to a BMG plate reader programmed to incubate and measure the OD600_nm_ of each well, as an indicator of *P. tolaasii* 2192^T^ growth, immediately, and then every 30 minutes for 24 hours. *B. bacteriovorus* HD100 alone does not produce an OD600_nm_ value due to its small cell size [48].

### Testing the effect of *B. bacteriovorus* predation of *P. tolaasii* on brown blotch lesion intensity on infected mushrooms

Button mushrooms (*Agaricus bisporus*) used in this experiment were sourced from a supermarket and thus were from a non-sterile setting. Wearing gloves to avoid hand contamination, mushrooms were gently wiped clean with laboratory tissue to remove any attached compost and excess surface moisture, but allow the mushroom epidermis to remain intact. Stipes were trimmed flat with a sterile scalpel blade, and each mushroom was placed, pileus side up, in a sterile 50 ml skirted Falcon tube. Bacterial preparations were grown in liquid culture as before, but concentrated before use, by centrifugation in Falcon tubes at 5200 rpm, 20 min at 25°C in a Sigma 4 K15 centrifuge and resuspension in King’s Medium B to the appropriate concentration (which was checked by viable counting after the experiments). (The *P. tolaasii* 2192^T^ produced only beige smooth colonies on the King’s Medium B, after 24 hour incubation at 29°C.) Concentrations used in the 15 μl applications to the mushrooms were as follows: *P. tolaasii* 2192^T^ (1.7 × 10^6^ Colony Forming Units, CFU, 15 μl^−1^), *B. bacteriovorus* HD100 (2.9 × 10^6^ Plaque-Forming Units, PFU, 15 μl^−1^) and King’s Medium B were applied directly to the mushroom pileus in one of 5 pairwise combinations for the experiment in Figure 2 (see Table 3 below). In later experiments other concentrations of bacteria were used as described.

**Table 3:** Treatment conditions applied to mushroom pilei

**Condition**	**Addition 1 (in 15 μl)**	**30 min, 21ËšC**	**Addition 2 (in 15 μl)**	**48 h, 29°C**
King’s Medium B control	King’s Medium B broth	→	King’s Medium B broth	→
*B. bacteriovorus* alone	*B. bacteriovorus* HD100	→	King’s Medium B broth	→
*P. tolaasii* alone	*P. tolaasii* 2192^T^	→	King’s Medium B broth	→
*B. bacteriovorus* before *P. tolaasii*	*B. bacteriovorus* HD100	→	*P. tolaasii* 2192^T^	→
*B. bacteriovorus* after *P. tolaasii*	*P. tolaasii* 2192^T^	→	*B. bacteriovorus* HD100	→

Details of the 5 pairwise combinations of *B. bacteriovorus* HD100, *P. tolaasii* 2192^T^ and King’s Medium B added to *Agaricus bisporus* mushrooms to test the effect of *B. bacteriovorus* predation of *P. tolaasii* on affected mushroom brown blotch lesion intensity.

Mushrooms were incubated statically at 29°C, in capped Falcon tubes for 48 hours, after which brown blotch lesions appeared on *P. tolaasii* 2192^T^ infected samples. Lesions were photographed using a Canon PowerShot A620 digital camera and tripod in a containment hood, with the same standard lighting for each photograph. The aperture was set to F = 5.6 and shutter speed was set to 1/60 sec, to give a good light exposure (±0 Exposure Value units). The lesion intensity on each mushroom was analysed using ImageJ analysis software (http://rsbweb.nih.gov/ij/): Image J converted each image to 8-bit grayscale, assigning a value of 0–255 to each pixel; the area of mushroom inoculated was selected and the average grayscale value for each pixel (the Pixel Value, PV), was calculated. On this scale, 0 = black and 255 = white, and so the data were transformed using the formula 1/PV to invert the scale, so that darker lesions give higher intensity values. These transformed data are displayed in Figures 2 and 4.

### Visualising *B. bacteriovorus* and *P. tolaasii* interactions on the mushroom surface

Mushrooms under each of the five treatment conditions detailed in Table 3 were visualised using Scanning Electron Microscopy. Preparation of mushroom samples for imaging was as follows: Samples of mushroom pileus surface tissue W 5 mm × L 5 mm × D 2 mm were cut and stored in 70% ethanol. They were then dehydrated through a graded series of ethanol concentrations (fresh 70% ethanol, followed by 90% ethanol, and finally 2 changes of 100% ethanol) and dried using a Polaron E3000 Critical Point Dryer. The dried samples were mounted onto aluminium stubs using silver paint, and the stubs were gold coated (~10 μm thickness) using a Polaron E5100 SEM Coating Unit. The samples were viewed and photographed under a JEOL JSM 840 Scanning Electron Microscope at 20 kV. Images were false-coloured in Adobe Photoshop by selecting *P. tolaasii* 2192^T^ and *B. bacteriovorus* HD100 cells and using the ‘Colorize’ function in the ‘Hue/Saturation’ tool. A pale yellow colour was selected for *P. tolaasii* to provide optimum contrast to the mushroom surface, and blue gave a sharp contrast for the *B. bacteriovorus.*

### Enumerating *P. tolaasii* recovered from infected mushroom tissue

Mushrooms were pre-treated using methods as above; *B. bacteriovorus* HD100 was applied at either 2.9 × 10^6^ or 1.4 × 10^7^ PFU 15 μl^−1^ before 1.7 × 10^6^*P. tolaasii* 2192^T^ in 15 μl. Mushroom lesions were photographed in a class II containment hood after 48 hours, as above, and lesion intensities were analysed using ImageJ analysis software. Lesion tissue from each mushroom was then cut out using a sterile scalpel blade. Tissue samples were weighed and homogenised in sterile 2 mM CaCl_2_ 25 mM HEPES pH 7.6 buffer (1 ml Calcium HEPES/0.04 g lesion tissue) using separate glass pestle and mortar sets, (pre-cleaned with ethanol and dried), for samples under each of the different treatment combinations. *P. tolaasii* 2192^T^ CFU recovered from each sample were enumerated by serial dilution and plating on King’s Medium B agar, incubated at 29°C for 15 hours. Characteristic smooth, beige colonies growing on King’s Medium B were counted and recorded as *P. tolaasii*.

### Identifying bacterial species co-isolated from some experimentally infected supermarket mushroom tissue

When King’s Medium B plates were examined and counted to enumerate *P. tolaasii* 2192^T^ from one batch of six mushrooms (two in each treatment group), a relatively high number of bacterial colonies, some of which were small and clumped together on the King’s B medium enumeration plates, were recovered from *P. tolaasii* 2192^T^ inoculated mushroom tissue pre-treated with *B. bacteriovorus* HD100 compared with tissue inoculated with *P. tolaasii* 2192^T^ alone. This suggested that other, possibly indigenous, bacteria were present, in addition to the added *P. tolaasii* 2192^T^ and *B. bacteriovorus* HD100. To test this, 20 single colonies were selected from the small clumped colonies recovered from mushroom tissue pre-treated with *B. bacteriovorus* HD100 at both 2.9 × 10^6^ and 1.4 × 10^7^ PFU ml^−1^ (taken from two mushrooms from each group). These were plated directly onto Coliform chromogenic agar (CCA) (Oxoid) and incubated at 29°C for 15 hours, along with a *P. tolaasii* 2192^T^ control, to distinguish between Pseudomonads and Coliforms. All of these small, clumped colonies were purple on CCA, indicating a different identity to *P. tolaasii* 2192^T^*,* which gave straw-coloured colonies on CCA. Total genomic DNA from each of 3 purple coliform isolates (hereafter referred to as Supermarket Mushroom Isolates 1, 2 and 3) was extracted using a Sigma DNA extraction kit and ‘universal’ 16 s ribosomal DNA primers (Table 2) were used in PCR reactions to amplify 16 s rDNA sequences which were sequenced by Source Bioscience Life Sciences, using the same primers. The resulting sequences were used to identify the closest match to the 16 s rDNA sequences of the isolates using the BLAST online tool, http://blast.ncbi.nlm.nih.gov/Blast.cgi.

## Competing interests

The authors declare that this work was funded in a joint project between Universities of Nottingham and Reading and BBSRC Rothamsted Experimental Research Station. These organizations do not have anything to gain financially from the publication of this manuscript. There are no competing financial or non-financial interests in the manuscript.

## Authors’ contributions

EBS carried out the *in vitro* predation assay and *in vivo* mushroom studies, participated in SEM imaging, carried out phylogenetic analyses, and drafted the manuscript. RWJ provided the *P. tolaasii* strain used in this study, and helped to draft the manuscript. SB was an undergraduate who participated in the *in vivo* mushroom studies. TS prepared mushroom samples for SEM, and carried out SEM imaging. RES conceived of the study and participated in its design and coordination, and helped to draft and edit the manuscript. All authors saw and approved a final edited version of the manuscript.
